# Serum Albumin and Body Weight as Biomarkers for the Antemortem Identification of Bone and Gastrointestinal Disease in the Common Marmoset

**DOI:** 10.1371/journal.pone.0082747

**Published:** 2013-12-06

**Authors:** Victoria K. Baxter, Gillian C. Shaw, Nathaniel P. Sotuyo, Cathy S. Carlson, Erik J. Olson, M. Christine Zink, Joseph L. Mankowski, Robert J. Adams, Eric K. Hutchinson, Kelly A. Metcalf Pate

**Affiliations:** 1 Department of Molecular and Comparative Pathobiology, The Johns Hopkins University School of Medicine, Baltimore, Maryland, United States of America; 2 Department of Biomedical Engineering, The Johns Hopkins University School of Medicine, Baltimore, Maryland, United States of America; 3 Department of Veterinary Population Medicine, University of Minnesota College of Veterinary Medicine, St. Paul, Minnesota, United States of America; University of Toronto, Canada

## Abstract

The increasing use of the common marmoset (*Callithrix jacchus*) in research makes it important to diagnose spontaneous disease that may confound experimental studies. Bone disease and gastrointestinal disease are two major causes of morbidity and mortality in captive marmosets, but currently no effective antemortem tests are available to identify affected animals prior to the terminal stage of disease. In this study we propose that bone disease and gastrointestinal disease are associated disease entities in marmosets and aim to establish the efficacy of several economical antemortem tests in identifying and predicting disease. Tissues from marmosets were examined to define affected animals and unaffected controls. Complete blood count, serum chemistry values, body weight, quantitative radiographs, and tissue-specific biochemical markers were evaluated as candidate biomarkers for disease. Bone and gastrointestinal disease were associated, with marmosets being over seven times more likely to have either concurrent bone and gastrointestinal disease or neither disease as opposed to lesions in only one organ system. When used in tandem, serum albumin <3.5 g/dL and body weight <325 g identified 100% of the marmosets affected with concurrent bone and gastrointestinal disease. Progressive body weight loss of 0.05% of peak body weight per day predicted which marmosets would develop disease prior to the terminal stage. Bone tissue-specific tests, such as quantitative analysis of radiographs and serum parathyroid hormone levels, were effective for distinguishing between marmosets with bone disease and those without. These results provide an avenue for making informed decisions regarding the removal of affected marmosets from studies in a timely manner, preserving the integrity of research results.

## Introduction

The common marmoset (*Callithrix jacchus*) is a non-endangered member of the New World nonhuman primate (NHP) family Callitrichidae native to northern and eastern Brazil. A small adult body size, short generation time, and low zoonotic disease risk compared to other NHPs make these monkeys attractive laboratory animals, and marmosets are commonly used in pharmacology, neuroscience, reproduction, obesity, immunology, and infectious disease studies [1], [2]. Marmosets have become increasingly popular as research models over the last few decades, with the number of publications rising forty-fold from the 1970’s to the 1990’s [3]. They are now the most common NHPs used in research in Europe [4], and their experimental use has been steadily increasing in North America since 1990 [3]. Nonprofit organizations designed to facilitate communication among researchers who work with marmosets, such as the European Marmoset Research Group and the Marmoset Research Group of the Americas, have formed in response to these developments [2], [3]. The frequent use of marmosets in research has resulted in the common marmoset being the fourth NHP (after the chimpanzee, macaque, and orangutan) and the first New World monkey to be selected for complete genome sequencing [5]. A thorough understanding of the normative care and biology of marmosets, including spontaneous diseases, is of great importance for obtaining and developing the highest standards of care for animals in captive colonies and thus preserving the health of these valuable models of human disease.

Gastrointestinal (GI) disease, including Marmoset Wasting Syndrome (MWS), and bone disease are endemic in many captive marmoset colonies and result in significant mortality. MWS, commonly referred to as “chronic wasting,” is one of the most important non-infectious marmoset diseases [6], with a 28–60% incidence in captive colonies [7], [8]. As described in multiple case reports, the disease is clinically characterized by impaired weight gain, muscle atrophy, alopecia (especially at the tail base), diarrhea, and weight loss; animals with the condition have been reported to lose as much as 25% to 50% of their body weight [7], [9]–[14]. On clinical bloodwork, animals with MWS have a number of abnormalities, including anemia, hypoproteinemia, hypoalbuminemia, and elevated serum alkaline phosphatase levels [6], [13], [14]. The gold standard for diagnosing MWS is through postmortem histologic examination of tissues, with chronic lymphoplasmacytic enterocolitis being the hallmark lesion [6], [8], [10], [12], [13], [15], [16]. Several etiologies for MWS have been proposed, including food allergies [17], [18], parasitism [19], [20], and autoimmune disease [21], but the cause of the condition remains unknown. Unfortunately there are no consistently effective treatments for halting the progression of or reversing the disease process, and due to a lack of proven antemortem diagnostic tools, affected marmosets are often not identified until the terminal stage of disease, at which point they are often already enrolled in research studies or breeding programs.

Another significant cause of mortality in captive marmoset colonies is bone disease, particularly metabolic bone diseases (MBDs). MBDs can be characterized by fibrous osteodystrophy (classic MBD), osteopenia, or rickets, and are caused by high bone turnover secondary to calcium-phosphorus imbalance, malabsorption, vitamin D deficiency, and/or excess parathyroid hormone (PTH) [22]. MBD has been recognized in NHP colonies for over 100 years, where it was originally referred to as “cage paralysis” or “cripples” [23]. Due to a dietary requirement of vitamin D3 and end organ resistance to vitamin D stimulation, New World monkeys are particularly susceptible [11], [24]–[27]. The incidence of disease has decreased significantly with the development of commercially available balanced diets containing adequate levels of vitamin D3 for captive New World primates; however bone disease is still reported sporadically in individual marmosets despite feeding these diets, with the underlying cause of disease unclear. Malabsorption of vitamin D and other nutrients, which may occur secondary to intestinal inflammation, is associated with an increased incidence of MBD [28], and Jarcho *et al* recently proposed that both MWS and MBD could be sequelae of the same pathologic process caused by impaired digestive efficiency [29].

Bone disease and GI disease, both individually and in combination, have devastating effects on marmoset health and the potential to hinder long-term research projects when diagnosed late in the clinical course of disease. We hypothesize that bone disease and GI disease are associated in marmosets, and that antemortem biomarkers indicative of GI health and bone metabolism correlate with histopathologic findings. Early and accurate identification of marmosets predisposed to or in the early stages of disease would preserve research integrity by allowing for the early exclusion of affected animals from long-term projects. In this study, we establish that bone and GI disease are associated in marmosets, and multiple antemortem biomarkers, including serum albumin, body weight, radiographs, and parathyroid hormone, can be used to distinguish between affected and unaffected individuals.

## Materials and Methods

### Ethics Statement

All experimental procedures were approved and overseen by the Institutional Animal Care and Use Committee of Johns Hopkins University. Strict adherence to the Guide for the Care and Use of Laboratory Animals by the National Institutes of Health, the Animal Welfare Act by the United States Department of Agriculture, and the Weatherall Report by the Medical Research Council was observed.

### Study Animals

All marmosets were housed in family units, in pairs, or singly as part of a large breeding and experimental colony at Johns Hopkins University, an Association for Assessment and Accreditation of Laboratory Animal Care International (AAALAC)-accredited institution. Animals were given ad libitum access to water and were fed a complete and balanced diet consisting of a custom homogenized blend of Teklad 8794N New World Primate Diet (Harlan Laboratories, Indianapolis, IN), Zupreem 9920.CS canned marmoset diet (Shawnee, KS), and Bio-Serv Newberne Hayes Vitamin Mix (San Diego, CA), supplemented with fruit and yogurt. Environmental enrichment in the form of visual and auditory contact with conspecifics, complex environments, and manipulanda were provided to all marmosets. Experienced animal care technicians observed animals at least once daily and more often if necessitated by experimental or medical need. The Johns Hopkins University Research Animal Resources veterinarians diagnosed, treated, and/or managed any medical illness or injury in the marmosets, and if euthanasia due to disease was warranted, marmosets were euthanized with an intravenous overdose of Euthasol (Virbac Corporation, Fort Worth, TX) under deep ketamine anesthesia. Of the 105 marmosets evaluated for this study, 62 marmosets served as colony breeders or were not on experimental study. The remaining 43 marmosets participated in neurobehavioral studies, of which 33 had surgically implanted headcaps [30]; five of the non-headcapped animals on study were maintained on a diet at approximately 90% of their free feeding weight as part of an experimental protocol [31]. At the end of experimental studies, marmosets were euthanized with a 200 mg/kg intraperitoneal overdose of Euthasol (Virbac Corporation, Fort Worth, TX) after being anesthetized with 40 mg/kg ketamine intramuscularly, and were perfused with 0.5 L of 0.1 M heparinized phosphate buffered saline followed by 1 L of 4% paraformaldehyde solution to preserve tissues. Animals included in bone-specific biomarker and weight analyses were all older than two years at death, consistent with physical and sexual maturity. Analyses were performed based on the availability of previously collected and recorded data, banked tissues, and/or stored serum samples, so each individual animal was not included in every comparison (Table S1).

### Histopathologic Disease Definitions

Bone and GI disease statuses for all animals in this study were determined by histopathologic analysis of postmortem tissues rather than the presence of clinical disease. Hematoxylin and eosin-stained slides from paraffin blocks containing bone and gastrointestinal tissue from 105 marmosets necropsied between October 1999 and February 2012 were examined by veterinary pathologists (Diplomates of the American College of Veterinary Pathologists). Animals were diagnosed as having abnormal bone if any of the following lesions were identified: abnormal growth plate (disorganized or abnormally oriented chondrocytes, thickened or fractured trabecular bones), abnormal cortices (thin – osteopenia, fractured without prior history of trauma, increased numbers of resorption cavities with increased osteoclastic +/– osteoblastic activity), and/or myelofibrosis. Animals were diagnosed as having GI disease if they had chronic mild inflammation in two or more segments or chronic moderate or severe inflammation in one or more segments of small or large intestine, including cecum. Animals with severe intestinal necrosis consistent with *Klebsiella* spp. infection were excluded from the study. Tissue samples that were of insufficient quantity or quality were designated nondiagnostic. Bone disease status and GI disease status for each animal were considered both in conjunction and independently during analyses.

### Clinical Records, Physical Exams, and Bloodwork

Data on weight and bloodwork parameters (including albumin) were retrospectively obtained from clinical and laboratory records from deceased marmosets and through veterinary physical exam of live marmosets. Terminal weight records were obtained from catalogued necropsy reports, and historically obtained bloodwork data were collected from clinical records. All live marmosets in the colony underwent a yearly physical exam while sedated with 20 mg/kg ketamine, which included body weight, superficial dental examination, and fur coat quality check. Blood was collected from the femoral vein, and a complete blood count and serum chemistry panel were performed using a Hemavet 950 hematology analyzer (Drew Scientific Inc, Oxford, CT) and vetACE chemical analyzer (Alfa Wassermann, West Caldwell, NJ), respectively. All bloodwork data used in this study were collected within one year of death, and in cases of multiple available records, the data collected closest to the date of death were included in analyses. Progressive weight data were collected by the lab as part of a standard experimental protocol from five animals that died due to natural causes and were diagnosed with GI disease +/– bone disease at necropsy. Weight data from five age- and gender-matched animals diagnosed with neither bone nor GI disease at necropsy were used for comparison. Data were graphed as percentage peak body weight versus days before death, and the slopes of the best-fit lines for each of the animals’ body weights were calculated.

### Radiographs

Marmosets were sedated with 20 mg/kg ketamine and were positioned in a standardized manner using a restraint board prior to taking ventrodorsal radiographs with the FCR XC-2 digital radiography system (Fujifilm, Valhalla, NY). A mammographic aluminum stepwedge with 9 sequential steps of radiodensity (Gammex, Inc, Middleton, WI) was included in all radiographs. Images were imported into an image analysis software package (Elements, Nikon Imaging Software). The distal 25% of both femurs were gated as regions of interest, and the bone radiodensity fraction (BRF) within the region of interest was determined using the stepwedge as a standard. These values ranged from zero to one, with a BRF closer to one representative of more radiopaque (dense) bone.

### Biochemical Markers

Serum markers of bone disease were measured using commercially available enzyme-linked immunosorbent assays (ELISA) for bone alkaline phosphatase (Quidel Corporation, San Diego, CA), parathyroid hormone (ALPCO Diagnostics, Salem, NH), and serum cross laps (Immunodiagnostic Systems, Scottsdale, AZ), while serum and fecal markers of gastrointestinal disease were measured using ELISAs for C-reactive protein (Life Diagnostics, West Chester, PA), secretory IgA (ALPCO Diagnostics, Salem, NH), and calprotectin (Buhlmann Laboratories, Schoenenbuch, Switzerland). Additional biomarkers specific for bone or GI disease were examined and found not to cross-react with marmoset samples (Table S2). Samples used had been previously banked or collected as part of annual clinical examination and stored at -80°C. Only samples collected within one year of death were used, and serum was thawed no more than twice.

### Statistics

Due to insufficient numbers of unaffected marmosets in some categories to prove normal data distribution, nonparametric statistical analyses were used. Two-tailed Mann Whitney U tests were used to compare disease and non-diseased groups, and Spearman’s rank (two tailed) was used to measure correlation between data. Two-tailed Fisher’s exact (FE) tests were used to calculate the odds ratio, sensitivity, specificity, positive predictive value (PPV) and negative predictive value (NPV). A *P* value of less than 0.05 was considered significant. All statistical analyses were performed using Graphpad Prism 6 (GraphPad Software, Inc.).

## Results

### Bone disease and GI disease are associated in marmosets

**Figure 1:**
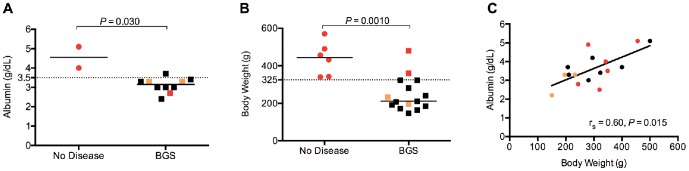
Serum albumin and body weight are decreased in marmosets with BGS. (A) Serum albumin and (B) body weight of marmosets diagnosed with BGS or no disease at necropsy. Solid horizontal lines represent median values for each group of data points, dotted horizontal lines denote cutoff values suitable to identify animals with BGS. (C) Correlation between body weight and albumin levels in individual animals. Solid black line represents best-fit line of the data points. Red data points denote animals with headcaps, gold data points denote animals on restricted diets.

Both bone disease and GI disease are common causes of morbidity and mortality in captive marmoset colonies, and we sought to elucidate whether diseases in these two organ systems were associated. We reviewed 105 marmoset necropsy records, with both bone and GI tissue available from 53 of these cases. Twenty-nine of these marmosets (55%) had both bone disease and GI disease, three (6%) had bone disease only, twelve (22%) had GI disease only, and nine (17%) had neither bone nor GI disease (Figure 1). Bone disease and GI disease were associated in individual marmosets, with a 7.25 odds ratio of concurrent bone and GI disease as opposed to bone or GI disease alone (FE *P* = 0.0070, 95% confidence interval: 1.67 to 31.53). As bone and GI disease are associated in our marmoset colony, we defined concurrent bone and GI disease as “bone and gastrointestinal syndrome” (BGS).

### BGS can be identified antemortem using serum albumin and/or body weight

Despite numerous case reports describing the clinical course of disease and postmortem findings of either bone or GI disease in marmosets [6], [10], [12], [13], [15], [16], [24], [32], parameters for antemortem identification of either individual disease or BGS as a syndrome are lacking. We examined whether commonly collected body weight and/or clinical pathology data could identify marmosets with BGS. Of the parameters measured by complete blood count and serum clinical chemistry tests, we selected hematocrit, platelet count, serum aspartate aminotransferase, alkaline phosphatase, total protein, serum albumin, and serum calcium to examine for predictive potential because alterations in these parameters have previously been described in clinical case reports of either MBD or MWS (Table S3) [6], [13], [24], [33].

Of the clinical chemistry parameters examined in a subset of 31 marmosets, serum albumin differed significantly between animals with BGS compared to unaffected animals (Mann-Whitney [MW] *P* = 0.030, Figure 1A). Normal albumin levels in common marmosets range from 3.5 to 5.1 g/dL [34], [35]; marmosets with BGS had a median serum albumin level of 3.2 g/dL, versus a median of 4.6 g/dL in unaffected animals. Serum albumin was also significantly decreased in animals with bone disease alone compared to animals without bone disease (MW *P* = 0.00010, Figure S1A). Additionally, a serum albumin level of less than 3.5 g/dL could distinguish animals with BGS from non-diseased animals, animals with bone disease from animals without bone disease, and animals with GI disease from animals without GI disease (FE *P* = 0.046, *P* = 0.0015, and *P* = 0.044 respectively, Table 1).

**Table 1:** Physical exam, clinical chemistry, and biochemical marker parameters can identify live marmosets with bone and/or GI disease.

	Test	Sensitivity	Specificity	PPV	NPV	*P* value
**Combined Disease (BGS) vs No Disease**	Serum Albumin < 3.5 g/dL	90%	100%	100%	67%	0.046
	Body Weight < 325 g	87%	100%	100%	75%	0.00050
	Serum Albumin < 3.5 g/dL or body weight < 325 g	100%	100%	100%	100%	0.036
	Weight loss > 0.05% peak weight per day	100%	100%	100%	100%	0.018
**Bone Disease vs No Bone Disease**	Serum Albumin < 3.5 g/dL	83%	100%	100%	75%	0.0015
	Body Weight < 325 g	81%	60%	68%	75%	0.029
	Serum Albumin < 3.5 g/dL or body weight < 325 g	86%	67%	86%	67%	*0.18*
	Weight loss > 0.05% peak weight per day	100%	83%	75%	100%	0.048
	BRF < 0.5	83%	100%	100%	80%	0.048
	PTH > 600 pg/mL	75%	100%	100%	73%	0.0014
**GI Disease vs No GI Disease**	Serum Albumin < 3.5 g/dL	70%	75%	88%	50%	0.044
	Body Weight < 325 g	75%	93%	96%	59%	< 0.00010
	Serum Albumin < 3.5 g/dL or body weight < 325 g	92%	100%	100%	80%	0.0027
	Weight loss > 0.05% peak weight per day	100%	100%	100%	100%	0.0079

=  positive predictive value, NPV  =  negative predictive value, BRF  =  bone radiodensity frequency, PTH  =  parathyroid hormone. PPV

*P* values calculated by Fisher’s exact test.

Italicized *P* values denote non-significant values (*P* ≥ 0.5).

Serum calcium is bound by albumin in the blood, and values reported in serum chemistry panels can therefore be confounded by alterations in serum albumin level [36]. Mathematical correction (corrected calcium [mg/dL]  =  measured total Ca^2+^ [mg/dL] + 0.8 * [4.3 - serum albumin {g/dL}]) can compensate for this artifact to obtain a more accurate assessment of serum calcium levels [37]. There was no significant difference in corrected serum calcium levels in bone disease marmosets compared to marmosets without bone disease (median 9.2 mg/dL vs 9.3 mg/dL, MW *P* = 0.26), though uncorrected serum calcium values were lower in marmosets with bone disease compared to animals with no bone disease secondary to significantly lower levels of albumin (median 8.2 mg/dL vs 9.1 mg/dL, MW *P* = 0.011, Table S3).

In addition to hematology and serum chemistry parameters, we examined whether physical exam data, such as body weight, could be used to identify animals with BGS. Adult body weights in captive marmoset colonies range from 250 to 600 g, with most animals weighing 350 to 400 g [38]. In our colony, examination of terminal body weights of 50 adult marmosets revealed that animals with BGS had significantly lower body weights (median 211 g), compared to animals with no disease (median 444 g, MW *P* = 0.0010, Figure 1B). Animals with bone disease had significantly lower body weights than animals with no bone disease (median 222 g versus 340 g, MW *P* = 0.013, Figure S1B). Additionally, consistent with previous reports of marmosets with MWS [6], [7], [12], animals with GI disease had significantly lower terminal body weights than animals with no GI disease (median 247 g versus 410 g, MW *P* < 0.00010, Figure S1C). Using 325 g as a cutoff value, we could significantly differentiate between disease states, with animals of body weight less than 325 g four times more likely to have BGS than heavier marmosets (relative risk [RR]  = 4.00, FE *P* = 0.00050, 95% confidence interval: 1.20 to 13.29, Table 1). Similar increases in risk for bone disease and GI disease were observed in animals with a body weight of less than 325 g (RR = 2.74, FE *P* = 0.029 for bone disease and RR = 2.36, FE *P* < 0.0001 for GI disease). Serum albumin level and body weight correlated within individual marmosets (Spearman ρ = 0.60, *P* = 0.015, Figure 1C). When considered in tandem as a biomarker panel, either a serum albumin level of less than 3.5 g/dL or a body weight of less than 325 g could significantly differentiate between affected and unaffected animals with BGS and GI disease, with a 100% sensitivity, specificity, PPV, and NPV in distinguishing BGS from unaffected animals (Table 1).

Negative correlations between marmoset age and serum albumin and body weight have been previously reported, with older adult marmosets generally having lower serum albumin levels and lower body weights compared to younger adult marmosets [38], [39]. No significant relationship was found between age and serum albumin (Spearman ρ = –0.051, *P* = 0.79) or age and body weight (Spearman ρ =  –0.046, *P* = 0.75) in our marmosets. Therefore, age was found to not be a confounder in the analysis of these parameters in marmosets with bone and/or GI disease, though our data does contrast with the results of previous studies.

Thirty-one percent of the marmosets included in this investigation were involved in neurobehavioral research that required the use of headcaps (identified by red data points in Figure 1), and 5% were at some point on diets targeted to maintain them at 90% of their free feeding weight as part of an experimental protocol (identified by gold data points in Figure 1). To ensure that inclusion of these animals did not affect our results and conclusions, we additionally analyzed the data while considering the animals with headcaps or on restricted diets. There was no significant difference in serum albumin level between animals with and animals without headcaps (median 3.8 g/dL vs 3.3 g/dL, MW *P* = 0.50), but headcapped marmosets, regardless of disease status, had significantly higher body weights than non-headcapped marmosets (median 395 g vs 210 g, MW *P* < 0.0001). The headcaps used by the lab weigh 17.1 g +/– 1.6 g, and subtraction of this weight from the body weight of headcapped animals did not significantly alter the difference in body weight between BGS animals and unaffected animals (MW *P* = 0.0016). Therefore, the marmosets with headcaps were generally more robust than marmosets without headcaps; this could be because the experimental laboratory selected larger and healthier-appearing animals from the colony for experimental study or, less likely, because the headcaps somehow improved the condition of the animals. Statistical analyses comparing serum albumin levels and body weights in BGS animals versus unaffected animals could not be performed with headcapped animals excluded due to insufficient numbers of non-diseased marmosets. Neither albumin levels nor body weight significantly differed with the exclusion of animals on restricted diets (MW *P* = 0.044 and *P* = 0.0020, respectively). Because the presence of a headcap was not associated with either decreased albumin or decreased body weight, and because exclusion of animals on a restricted diet did not significantly impact our results, we chose to keep these marmosets in our analyses.

### Progressive weight trends predict development of BGS

**Figure 2:**
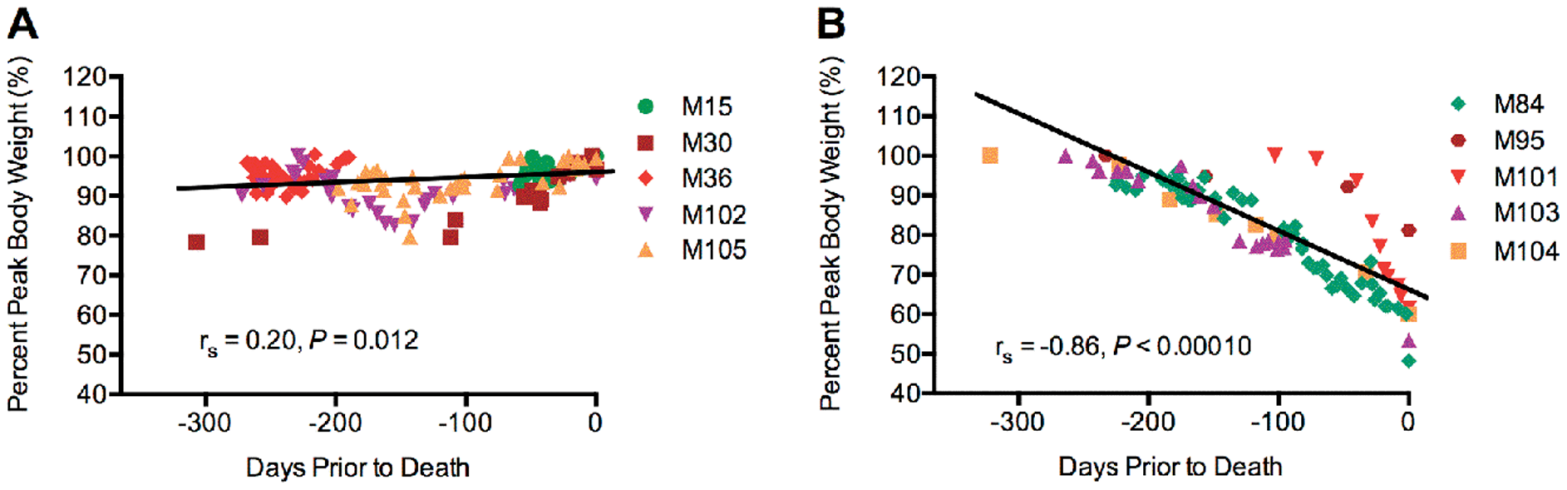
Bone and/or GI disease can be predicted by weight trends. Percent of peak body weight of five unaffected (A) and five marmosets with GI +/– bone disease (B) for the year prior to death. Colors represent data points from individual animals, black lines represent best-fit line of the data points.

While body weight and serum albumin provide a way to identify BGS prior to death, we wanted to identify a marker that could predict development of disease well before the terminal stage. Multiple sequential weight data were collected from five animals diagnosed with GI +/– bone disease at necropsy and five unaffected animals. Peak weight of each animal was determined, percentage of peak weight was calculated for all data available one year prior to death, and data were graphed as percentage peak body weight versus days before death. (Figures 2A and 2B).

Marmosets with GI +/– bone disease trended towards lower peak weights than unaffected animals (MW *P* = 0.056, Table 2), and at death, diseased animals were at a significantly lower percentage peak body weight (60%) compared to unaffected animals (99%, MW *P* = 0.0079, Table 2). Affected animals lost weight at a rate of 0.17% of peak body weight per day, or 1.2% per week. In contrast, unaffected animals maintained body weights in the year leading to death, with an average gain of 0.036% body weight per day. Loss of greater than 0.05% body weight per day significantly distinguished between affected and unaffected animals a median of 264 days before they reached the terminal stage of disease (FE *P* = 0.018, Table 1). Therefore, progressive body weight trends can be used to predict which animals will be affected with bone and/or GI disease.

**Table 2:** Progressive weight characteristics of marmosets affected or unaffected with GI +/– bone disease.

	Affected	Unaffected	P value
**Number of Subjects**	5	5	
**Male:Female Ratio**	3:2	2:3	
**Age at Death (Years)**	5.0 (2.2 8.6)	4.0 (2.8, 4.8)	*0.46*
**GI Disease:No GI Disease Ratio**	5:0	0:5	
**Bone Disease:No Bone Disease Ratio**	3:1*	0:5	
**Peak Body Weight (g)**	386 (304, 462)	484 (386, 569)	*0.056*
**% Peak Body Weight Lost at Death**	39.9 (23.5, 54.7)	0.5 (–1.3, 5.1)	0.0079
**% Weight Change per Day** **	–0.17 (–0.19, –0.14)	+0.036 (0.015, 0.058)	0.0079

Data presented as median values with parentheses following denoting 95% confidence intervals of the mean.

Italicized *P* values denote non-significant values (*P* ≥ 0.5).

* Bone tissue slides from one affected marmoset were nondiagnostic.

** Percent weight change per day represented as the median slope and 95% confidence interval of that slope.

Two marmosets included in the above weight trend analyses, M84 and M104, were initially kept on diets targeted to maintain them at 90% of their free feeding weight as described above until four months prior to death; when persistent weight loss was noted, the diets were discontinued. The average percent peak weight loss per day actually increased following termination of the restricted diet for each animal (0.068% vs 0.24% for M84 and 0.094% vs 0.18% for M104, during and after cessation of the defined diet, respectively). Therefore, the restricted diet did not accelerate weight loss, and return to a free choice-feeding regimen did not halt, slow, or reverse the progression of disease.

### Quantitative radiography and serum PTH can identify marmosets with bone disease

**Figure 3:**
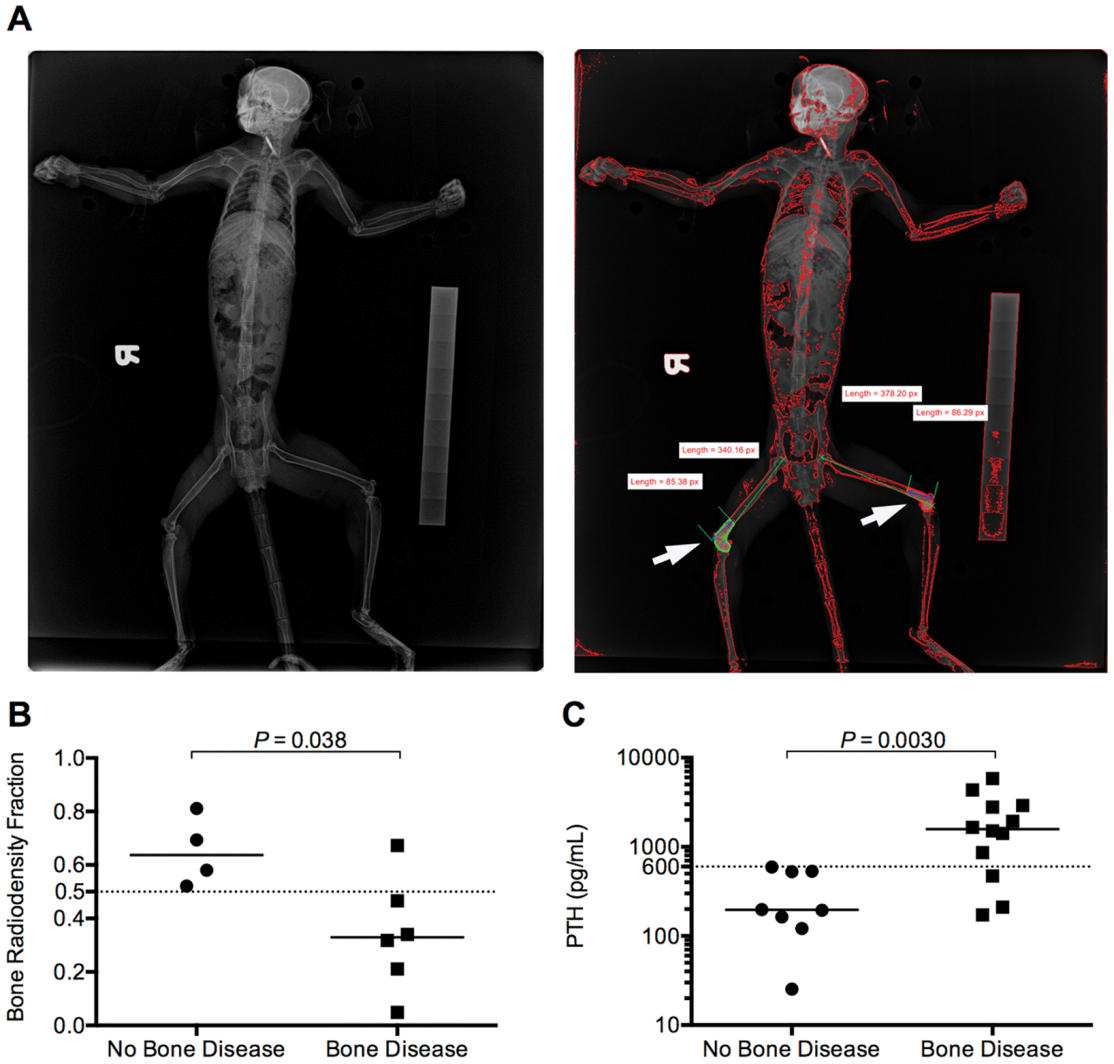
Skeletal system-specific tests can identify bone disease in marmosets. (A) Example of the initial radiographic image (left panel) and analyzed image (right panel) of an animal with bone disease, with BRF calculated from the distal quarter of each femur (white arrows). (B) BRF values in marmosets diagnosed with bone disease or with no bone disease at necropsy. (C) PTH levels in marmosets diagnosed with bone disease or with no bone disease at necropsy. BRF  =  bone radiodensity fraction, PTH  =  parathyroid hormone. Solid horizontal lines represent median values for each group of data points, dotted horizontal lines denote cutoff values to distinguish animals with bone disease.

We examined whether digital radiographs could quantify bone density and identify marmosets with bone disease. As part of our annual physical examination of the colony, ventrodorsal radiographs were taken with a digital radiography system, and the bone radiodensity fraction (BRF) from the images was determined using a stepwedge standard (Figure 3A). Marmosets with bone disease at necropsy had significantly lower BRFs compared to marmosets with no bone disease (MW *P* = 0.038, Figure 3B). Furthermore, a BRF of less than 0.5 significantly distinguished bone disease animals from animals without bone disease (Table 1). Insufficient numbers of animals were available to evaluate the utility of quantitative radiographs in distinguishing marmosets with GI disease from marmosets without GI disease or marmosets with BGS from unaffected marmosets.

Biochemical parameters specific to either the skeletal or the GI system were also examined as potential antemortem tests for BGS. Three serum markers specific for disease of the skeletal system were examined: bone alkaline phosphatase (BAP), parathyroid hormone (PTH), and carboxy-terminal collagen crosslinks (serum cross laps or SCL). BAP is an enzyme produced by osteoblasts and osteoclasts that is used as an indicator of general bone turnover [40], PTH is a calcium homeostasis-regulating hormone used to gauge calcium imbalance [41], and SCL is a collagen degradation product that indicates bone resorption [42]–[44]. PTH was significantly higher in marmosets with bone disease (MW *P*  =  0.0030, Figure 3C), and a PTH value of greater than 600 pg/mL could significantly differentiate animals with bone disease from animals without bone disease (Table 1). Serum BAP and SCL failed to distinguish affected from unaffected marmosets (Figures S2A and S2B); small sample size of unaffected animals precluded evaluation of these parameters for BGS or GI disease.

One serum and two fecal biochemical parameters specific for disease of the GI system were examined. C-reactive peptide (CRP) is a serum acute phase inflammatory protein that is used to detect systemic inflammatory disease, including GI disease, in both humans and nonhuman primates [45], [46] (Figure S2C), secretory IgA (sIgA) is a component of mucosal immunity that can serve as a fecal indicator of infectious enterocolitis [47]–[49] (Figure S2D), and calprotectin is a fecal marker produced by neutrophils used to detect irritable bowel syndrome [45], [50] (Figure S2E). None of these biomarkers were significantly altered in marmosets with BGS, bone, or GI disease.

## Discussion

Marmosets are commonly employed for long-term research studies, and development of confounding disease after a study is underway results in a considerable waste of effort and funds. Early identification of diseased animals prior to the terminal stage could prevent some of these losses and allow labs to remove animals from study or relegate them to acute procedures. Bone disease and GI disease are two conditions with unknown causes commonly found in captive marmosets, and in the present study we demonstrate that bone and GI disease are associated. We also establish retrospectively that low body weight (< 325 g) and low serum albumin (< 3.5 g/dL) are sufficient to identify animals affected with BGS, and progressive weight loss of more than 0.05% body weight per day is predictive for the later development of disease. We furthermore show that quantitative analysis of digital radiographs and serum PTH levels can be used to identify marmosets with bone disease.

The power of this panel of biomarkers lies in its accessibility to the research, zoo, and veterinary communities. The equipment necessary to measure body weight, collect blood, and take radiographs is commonly found in animal facilities, and the tests are economical to perform. When used together, body weight and serum albumin offer a powerful method for identifying marmosets with BGS or GI disease, with 100% PPV and NPV for BGS.

Body weight has proved to be a strong indicator of affected animals and an effective predictor of disease development. Weight loss has been identified as characteristic of MWS, but other than a comment in one study of a marmoset with osteomalacia [27], low body weight has not generally been associated with bone disease in marmosets. However, higher body weights in marmosets have been associated with increased bone mineral density [51]. In the present study we found low body weight to be a characteristic of animals with concurrent bone and GI disease, indicating weight can serve as an antemortem indicator for BGS. Additionally, weight loss of greater than 0.05% peak body weight per day can identify marmosets with bone and/or GI disease several months before death, providing a means of predicting which animals will develop disease.

Marmosets with BGS or bone disease possessed considerably lower serum albumin levels compared to unaffected animals, and a 3.5 g/dL cutoff could significantly distinguish between affected and unaffected animals. Hypoalbuminemia has been reported in humans with osteopenia [52] and has been associated with increased disease severity and higher mortality in both human and canine patients with inflammatory bowel disease [53]–[56]. Additionally, low serum albumin has been found to be a risk factor for low bone mineral density, a recognized complication of Crohn’s disease in humans [57]. While hypoalbuminemia has previously been reported in marmosets with MWS, we found that marmosets diagnosed with bone disease at necropsy also possessed lower serum albumin levels compared to animals without bone disease. This association has not previously been reported and further supports an association between bone disease and GI disease in marmosets. Serum calcium levels were lower in marmosets with bone disease, but once those levels were corrected for low albumin, the difference was no longer significant. While it was not performed in this study due to a lack of a sufficient volume of appropriate samples, future measurement of biologically active ionized calcium would provide more insight into the status of calcium homeostasis in marmosets with bone and/or GI disease [58], [59].

We additionally aimed to evaluate conventional radiography as a quantitative method to evaluate bone density for the identification of marmosets with bone disease. Radiological imaging provides a way to evaluate the skeletal system in a live animal. Skeletal integrity in marmosets has been evaluated by several modalities both ante- and postmortem, including conventional X-ray, computed tomography (CT), and dual energy X-ray absorptiometry (DEXA) [29], [43], [51], [60], [61]. Reports have focused on the density of specific bone regions, such as the proximal tibia, proximal femur, or lumbar vertebrae to draw conclusions on the bone quality of the marmoset as a whole. We chose to use the distal femur in our assessment because radiographs of the region can be taken in a live animal, it is relatively easy to position the leg for a consistent mediolateral view, and that specific area of the bone lacks significant muscle mass that can confound bone density evaluation. While it is well-established that a considerable percentage of bone must be lost before it becomes evident on a conventional radiograph [22], and many other imaging modalities are more sensitive in detecting changes in bone integrity, many facilities housing marmosets do not have access to or the financial resources to afford these advanced imaging techniques. However, most institutions with marmoset colonies have access to a digital X-ray system. Therefore, quantitative assessment of bone density using conventional radiography provides a relatively inexpensive, yet still accessible and effective method to evaluate skeletal integrity and identify marmosets with bone disease.

We originally evaluated a number of biochemical parameters specific for either bone or GI disease in humans, such as serum BAP, PTH, SCL, CRP, fecal sIgA, and fecal calprotectin, to determine if they could identify marmosets with BGS better than the more general measures of body weight, albumin or radiographs. Of the tests examined, none of these markers proved sufficient to identify BGS, and only PTH levels were found to significantly differ between marmosets with bone disease and without bone disease. PTH increases calcium release from bone into the blood through indirect stimulation of osteoclasts, making it a good indicator of bone turnover [41], and several disorders, including vitamin D deficiency, are characterized by elevated PTH [62]–[64]. Based on our findings, increased PTH levels may be used to distinguish marmosets with bone disease from unaffected marmosets. Re-evaluation of these serum and fecal biochemical markers with a larger cohort of marmosets may reveal diagnostic value.

While investigating the mechanism of BGS is beyond the scope of this project, our findings support an association between bone and GI disease, with marmosets in our colony 7.25 times more likely to have lesions in both skeletal tissue and GI tissue than in only one tissue or the other. Such a link between bone and GI disease supports the pathogenesis recently proposed by Jarcho *et al*., where GI inflammation leads to malabsorption of critical nutrients, including those important for bone homeostasis [29]. This then could lead to weight loss and muscle wasting typical of MWS and decreased bone density characteristic of MBD. Our finding that bone disease and GI disease are associated in marmosets provides additional evidence for a shared pathologic process, and the presence of low albumin levels in marmosets with BGS or bone disease support a malabsorptive process in the pathogenesis of bone disease. Longitudinal studies are warranted to further establish the pathophysiological link between bone disease and GI disease.

The widespread prevalence of this spontaneous disease in captive marmosets presents the opportunity to evaluate BGS as a potential model for human disease, similar to how another New World monkey, the cotton top tamarin (*Saguinus oedipus*), is used as a model for ulcerative colitis and colonic adenocarcinoma [65]–[67]. Biochemical markers for inflammatory bowel disease (pANCA and ASCA) [68] and celiac disease (anti-tissue transglutaminase antibody) [21], [69] were examined in our marmosets in an attempt to define the nature of BGS, but unfortunately the marmosets’ serum did not cross-react with the human diagnostic tests (Table S2). However, further examination of bone and gastrointestinal biomarkers in these animals may reveal how BGS in marmosets may be used as a potential model for human conditions.

At this time, bone and/or GI disease in marmosets remain untreatable. If GI disease truly precedes bone disease in marmosets as we suspect, early identification of affected animals may facilitate treatment and prevention of BGS. Several accounts report on therapeutic interventions that have reversed progression of MWS [9], [14], [17], [18], [20], but none of them have worked consistently. This could be because marmosets are diagnosed with bone disease and/or GI disease at a point beyond which treatments could potentially halt or reverse the disease process. Our tests may therefore be used to identify marmosets earlier in the progression of disease so that novel therapies can be evaluated. However, prospective studies are needed to validate the utility of these biomarkers in predicting and diagnosing disease.

In conclusion, we have found that bone disease and GI disease are indeed associated in marmosets, and the noninvasive antemortem tests of serum albumin and body weight can be used to identify affected animals prior to the terminal stage, especially when used in tandem. Furthermore, progressive weight trends can predict which animals will develop BGS prior to the terminal stage of disease, allowing for removal of these animals from experimental studies before significant long-term investments have been made. The inciting factor(s) that cause BGS remain unknown, and at this time no consistent, effective therapies are available to slow or reverse the disease process. Now that reliable antemortem biomarkers for BGS have been identified, further examination of the pathogenesis of BGS and the marmoset’s potential as a model for human bone and/or GI disease is warranted.

## Supporting Information

Figure S1 **Antemortem tests can be used to distinguish diseased and non-diseased animals.** Serum albumin (A) of marmosets diagnosed with bone disease or no bone disease at necropsy. Body weights of marmosets diagnosed with bone disease or no bone disease (B) and GI disease or no GI disease (C) at necropsy. Solid horizontal lines represent median values for each group of data points, dotted horizontal lines denote Fisher’s Exact cutoff values.
(TIFF)

Figure S2 **Biochemical biomarkers examined as potential diagnostic tests for bone disease and GI disease.** Antemortem BAP (A) and SCL (B) levels in marmosets diagnosed with bone disease or with no bone disease at necropsy. CRP levels (C), sIgA (D), and calprotectin (E) in marmosets diagnosed with GI disease or with no GI disease at necropsy. Solid horizontal lines represent median values for each group of data points.
(TIF)

Table S1 **Demographics, disease status, and analyses for each marmoset used in the study.**
(DOC)

Table S2 **Biomarkers found to not cross react with marmoset samples.**
(DOC)

Table S3 **Bloodwork parameters examined in identifying disease in marmosets.**
(DOC)
