# Supplementary figures and images for: Serum Albumin and Body Weight as Biomarkers for the Antemortem Identification of Bone and Gastrointestinal Disease in the Common Marmoset
Source: PLoS One. 2013 Dec 6;8(12):e82747. doi: 10.1371/journal.pone.0082747 (PMC3855796; doi:10.1371/journal.pone.0082747)

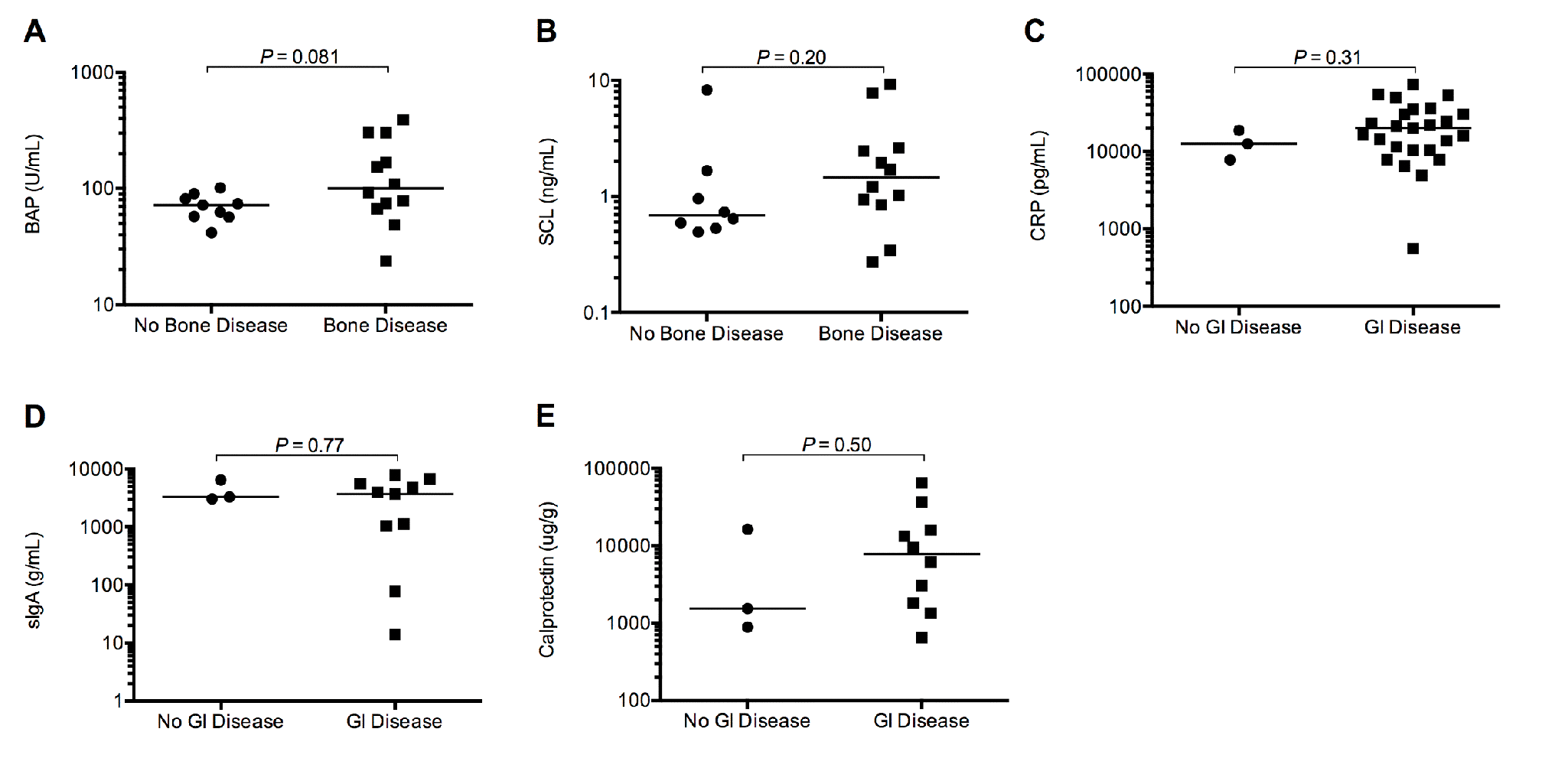

Supplement: Figure S2 — Biochemical biomarkers examined as potential diagnostic tests for bone disease and GI disease. Antemortem BAP (A) and SCL (B) levels in marmosets diagnosed with bone disease or with no bone disease at necropsy. CRP levels (C), sIgA (D), and calprotectin (E) in marmosets diagnosed with GI disease or with no GI disease at necropsy. Solid horizontal lines represent median values for each group of data points. (TIF) [file pone.0082747.s002.tif]
