# Supplementary material for: Serum Albumin and Body Weight as Biomarkers for the Antemortem Identification of Bone and Gastrointestinal Disease in the Common Marmoset
Source: PLoS One. 2013 Dec 6;8(12):e82747. doi: 10.1371/journal.pone.0082747 (PMC3855796; doi:10.1371/journal.pone.0082747)
Supplement: Table S1 — Demographics, disease status, and analyses for each marmoset used in the study. (DOC) [file pone.0082747.s003.doc]

**Table S1: Demographics, disease status, and analyses for each marmoset used in the study**

| **Marmoset ID** | **Sex** | **Age in Years at Death** | **Experimental Study** | **Cause of Death** | **Bone Disease?** | **GI Disease?** | **Analyses Included In** |
| --- | --- | --- | --- | --- | --- | --- | --- |
| M1 | M | 8 | Neurobehavioral Study - Headcap | Euthanized due to terminal illness | No | No | Dz Corr, Term Wt |
| M2 | M | 3 |  | Died without overt signs of clinical illness | ND | Yes | Term Wt |
| M3 | F | 2 |  | Euthanized due to MWS | No | Yes | Dz Corr, Term Wt |
| M4 | F | 8 |  | Euthanized due to MWS | Yes | Yes | Dz Corr, Term Wt |
| M5 | M | 9 |  | Not available | Yes | Yes | Dz Corr, Term Wt |
| M6 | M | 3 | Neurobehavioral Study - Headcap | Not available | ND | No | Bldwk, Term Wt |
| M7 | M | 0* |  | Euthanized due to terminal illness | No | ND |  |
| M8 | F | 4 | Neurobehavioral Study - Headcap | Died without overt signs of clinical illness | Yes | Yes | Dz Corr, Term Wt |
| M9 | M | 7 |  | Euthanized due to MBD | Yes | No | Dz Corr, Bldwk |
| M10 | M | 4 | Neurobehavioral Study - Headcap | Euthanized for experimental study | Yes | Yes | Dz Corr, Term Wt |
| M11 | M | 5 | Neurobehavioral Study - Headcap | Euthanized | ND | Yes | Term Wt |
| M12 | F | 8 |  | Euthanized due to terminal illness | ND | Yes | Bldwk, Term Wt |
| M13 | M | 2 |  | Euthanized for experimental study | No | No | Dz Corr |
| M14 | F | 8 |  | Not available | Yes | ND |  |
| M15 | M | 3 | Neurobehavioral Study - Headcap | Not available | No | No | Dz Corr, Term Wt, Prog Wt |
| M16 | M | 1* |  | Died without overt signs of clinical illness | ND | No |  |
| M17 | F | 1* |  | Died without overt signs of clinical illness | ND | No |  |
| M18 | M | 4 |  | Died without overt signs of clinical illness | ND | Yes |  |
| **Marmoset ID** | **Sex** | **Age in Years at Death** | **Experimental Study** | **Cause of Death** | **Bone Disease?** | **GI Disease?** | **Analyses Included In** |
| M19 | F | 2 |  | Died without overt signs of clinical illness | No | ND |  |
| M20 | F | 0* |  | Died during management of illness | ND | No |  |
| M21 | M | 5 |  | Died during management of MWS | ND | Yes |  |
| M22 | F | 0* | Neurobehavioral Study | Died without overt signs of clinical illness | ND | No | Ser Bio, Fecal Bio |
| M23 | F | 4 | Neurobehavioral Study - Headcap | Euthanized | ND | No | Term Wt |
| M24 | F | 7 |  | Euthanized due to MWS | ND | Yes | Bldwk, Term Wt |
| M25 | F | 11 |  | Euthanized due to MWS | ND | Yes |  |
| M26 | F | 5 | Neurobehavioral Study - Headcap | Euthanized for experimental study | ND | No | Term Wt |
| M27 | F | 4 | Neurobehavioral Study - Headcap | Euthanized due to terminal illness | ND | No | Bldwk |
| M28 | M | 7 |  | Died during management of MWS | ND | Yes | Term Wt |
| M29 | M | 5 |  | Euthanized for experimental study | ND | No |  |
| M30 | F | 5 | Neurobehavioral Study - Headcap | Euthanized for experimental study | No | No | Dz Corr, Term Wt, Prog Wt |
| M31 | M | 5 |  | Euthanized for experimental study | No | No | Dz Corr |
| M32 | F | 7 |  | Died without overt signs of clinical illness | Yes | Yes | Dz Corr, Term Wt |
| M33 | M | 1* |  | Euthanized due to MWS | ND | Yes |  |
| M34 | M | 1* |  | Died during management of illness | Yes | Yes | Dz Corr |
| M35 | F | 2 |  | Died during management of MWS | Yes | Yes | Dz Corr, Term Wt |
| M36 | F | 4 | Neurobehavioral Study - Headcap | Euthanized for experimental study | No | No | Dz Corr, Term Wt, Prog Wt |
| M37 | F | 4 |  | Died during management of MWS | ND | Yes |  |
| **Marmoset ID** | **Sex** | **Age in Years at Death** | **Experimental Study** | **Cause of Death** | **Bone Disease?** | **GI Disease?** | **Analyses Included In** |
| M38 | F | 1* |  | Euthanized due to terminal illness | Yes | Yes | Dz Corr |
| M39 | F | 4 |  | Euthanized due to MWS | Yes | Yes | Dz Corr, Term Wt |
| M40 | M | 2 |  | Euthanized due to MWS | ND | Yes | Term Wt |
| M41 | U | 1* |  | Died during management of MBD | Yes | ND |  |
| M42 | M | 1* |  | Died during management of illness | Yes | ND |  |
| M43 | F | 5 |  | Euthanized due to MWS | ND | Yes | Term Wt |
| M44 | M | 3 | Neurobehavioral Study - Headcap | Euthanized for experimental study | ND | Yes | Bldwk, Term Wt |
| M45 | F | 5 | Neurobehavioral Study - Headcap | Euthanized due to terminal illness | Yes | No | Dz Corr, Bldwk, Term Wt |
| M46 | M | 0* |  | Died during management of illness | Yes | ND |  |
| M47 | M | 2 |  | Euthanized due to MWS | ND | Yes | Bldwk |
| M48 | M | 1* |  | Euthanized due to terminal illness | ND | No |  |
| M49 | M | 0* |  | Euthanized due to terminal illness | Yes | ND |  |
| M50 | F | 2 | Neurobehavioral Study - Headcap | Euthanized due to MWS | ND | Yes | Term Wt |
| M51 | F | 0* |  | Died without overt signs of clinical illness | Yes | Yes | Dz Corr |
| M52 | M | 0* |  | Euthanized due to terminal illness | Yes | ND |  |
| M53 | M | 4 | Neurobehavioral Study - Headcap | Euthanized for experimental study | No | Yes | Dz Corr |
| M54 | F | 6 |  | Euthanized due to terminal illness | No | Yes | Dz Corr |
| M55 | M | 2 |  | Euthanized due to MWS | No | ND | Bldwk |
| M56 | F | 1* |  | Euthanized due to MWS | Yes | Yes | Dz Corr |
| **Marmoset ID** | **Sex** | **Age in Years at Death** | **Experimental Study** | **Cause of Death** | **Bone Disease?** | **GI Disease?** | **Analyses Included In** |
| M57 | F | 2 | Neurobehavioral Study | Euthanized due to terminal illness | ND | Yes | Bldwk |
| M58 | F | 0* |  | Euthanized due to MBD | Yes | No | Dz Corr, Ser Bio |
| M59 | M | 5 |  | Euthanized due to MWS | Yes | Yes | Dz Corr, Term Wt |
| M60 | M | 3 |  | Euthanized due to MWS | Yes | Yes | Dz Corr, Bldwk, Ser Bio |
| M61 | M | 6 |  | Died without overt signs of clinical illness | No | Yes | Dz Corr, Term Wt |
| M62 | F | 3 | Neurobehavioral Study - Headcap | Died without overt signs of clinical illness | No | ND | Bldwk |
| M63 | M | 2 |  | Died during management of MWS | Yes | Yes | Dz Corr |
| M64 | F | 5 | Neurobehavioral Study - Headcap | Euthanized due to MBD | Yes | Yes | Dz Corr, Bldwk, Ser Bio |
| M65 | M | 6 |  | Euthanized for experimental study | Yes | Yes | Dz Corr, Bldwk, Ser Bio |
| M66 | F | 5 | Neurobehavioral Study - Headcap | Euthanized for experimental study | ND | No | Term Wt |
| M67 | F | 5 | Neurobehavioral Study - Headcap | Euthanized due to MWS | No | Yes | Dz Corr, Ser Bio |
| M68 | F | 0* |  | Euthanized due to MWS | Yes | Yes | Dz Corr, Ser Bio |
| M69 | M | 3 | Neurobehavioral Study - Headcap | Died secondary to experimental procedure | ND | No | Term Wt |
| M70 | M | 2 | Neurobehavioral Study - Headcap | Died secondary to experimental procedure | ND | No | Term Wt |
| M71 | F | 6 | Neurobehavioral Study - Headcap | Euthanized | ND | No | Bldwk |
| M72 | M | 6 | Neurobehavioral Study - Headcap | Euthanized for experimental study | No | Yes | Dz Corr, Term Wt, Ser Bio |
| M73 | M | 7 | Neurobehavioral Study - Headcap | Died secondary to experimental procedure | No | Yes | Dz Corr, Term Wt |
| M74 | M | 3 |  | Euthanized for experimental study | ND | No | Bldwk |
| M75 | M | 0* |  | Died without overt signs of clinical illness | ND | Yes | Ser Bio |
| **Marmoset ID** | **Sex** | **Age in Years at Death** | **Experimental Study** | **Cause of Death** | **Bone Disease?** | **GI Disease?** | **Analyses Included In** |
| M76 | F | 7 |  | Euthanized due to MWS | ND | ND |  |
| M77 | F | 9 |  | Died during management of MWS | Yes | Yes | Dz Corr, Term Wt |
| M78 | M | 6 | Neurobehavioral Study - Headcap | Euthanized due to terminal illness | Yes | Yes | Dz Corr |
| M79 | M | 16 |  | Died during management of MWS | Yes | Yes | Dz Corr, Ser Bio |
| M80 | F | 6 | Neurobehavioral Study - Headcap | Euthanized due to terminal illness | No | No | Dz Corr |
| M81 | M | 1* |  | Died during management of MBD | Yes | Yes | Dz Corr, Ser Bio |
| M82 | F | U |  | Died without overt signs of clinical illness | Yes | Yes | Dz Corr, Ser Bio |
| M83 | M | 5 | Neurobehavioral Study - Headcap & Restricted Diet | Euthanized | ND | No | Term Wt |
| M84 | F | 5 | Neurobehavioral Study - Restricted Diet | Died during management of MWS | ND | Yes | Bldwk, Term Wt, Prog Wt, Ser Bio |
| M85 | F | 8 |  | Died without overt signs of clinical illness | ND | Yes | Term Wt |
| M86 | F | 6 | Neurobehavioral Study - Headcap | Euthanized due to terminal illness | No | Yes | Dz Corr, Term Wt, Ser Bio |
| M87 | F | 4 |  | Euthanized due to terminal illness | No | Yes | Dz Corr, Term Wt, Ser Bio, Fecal Bio |
| M88 | M | 6 | Neurobehavioral Study | Euthanized due to terminal illness | ND | Yes | Term Wt, Rad, Ser Bio, Fecal Bio |
| M89 | M | 5 | Neurobehavioral Study | Died during management of MBD | Yes | ND | Rad, Ser Bio, Fecal Bio |
| M90 | F | 6 | Neurobehavioral Study - Headcap | Euthanized due to terminal illness | ND | Yes | Bldwk, Ser Bio |
| M91 | M | 1* |  | Died without overt signs of clinical illness | ND | Yes |  |
| M92 | F | 1* |  | Died without overt signs of clinical illness | Yes | Yes | Dz Corr, Bldwk, Ser Bio, Fecal Bio |
| M93 | F | 4 | Neurobehavioral Study - Restricted Diet | Euthanized due to MWS | No | Yes | Dz Corr, Term Wt, Rad, Ser Bio |
| M94 | M | 4 |  | Died without overt signs of clinical illness | No | ND | Rad, Ser Bio, Fecal Bio |
| **Marmoset ID** | **Sex** | **Age in Years at Death** | **Experimental Study** | **Cause of Death** | **Bone Disease?** | **GI Disease?** | **Analyses Included In** |
| M95 | F | 3 |  | Died during management of illness | Yes | Yes | Dz Corr, Bldwk, Term Wt, Prog Wt, Rad, Ser Bio, Fecal Bio |
| M96 | F | 6 | Neurobehavioral Study - Headcap | Euthanized due to terminal illness | ND | Yes | Bldwk, Term Wt, Rad, Ser Bio, Fecal Bio |
| M97 | M | 6 | Neurobehavioral Study - Restricted Diet | Euthanized due to MWS | Yes | Yes | Dz Corr, Bldwk, Term Wt, Ser Bio, Fecal Bio |
| M98 | F | 3 | Neurobehavioral Study | Euthanized due to MWS | Yes | Yes | Dz Corr, Bldwk, Term Wt, Rad, Ser Bio, Fecal Bio |
| M99 | M | 4 |  | Euthanized due to terminal illness | No | Yes | Dz Corr, Bldwk, Ser Bio, Fecal Bio |
| M100 | F | 8 |  | Euthanized due to terminal illness | Yes | Yes | Dz Corr, Bldwk, Term Wt, Rad, Ser Bio, Fecal Bio |
| M101 | M | 3 | Neurobehavioral Study - Headcap | Died during management of illness | No | Yes | Dz Corr, Bldwk, Term Wt, Prog Wt, Rad, Ser Bio |
| M102 | F | 3 | Neurobehavioral Study - Headcap | Euthanized due to terminal illness | No | No | Dz Corr, Bldwk, Term Wt, Prog Wt, Rad, Ser Bio, Fecal Bio |
| M103 | M | 9 | Neurobehavioral Study | Died during management of MWS | Yes | Yes | Dz Corr, Bldwk, Term Wt, Prog Wt, Rad, Ser Bio, Fecal Bio |
| M104 | M | 7 | Neurobehavioral Study - Restricted Diet | Died during management of MWS | Yes | Yes | Dz Corr, Bldwk, Term Wt, Prog Wt, Rad, Ser Bio, Fecal Bio |
| M105 | M | 4 | Neurobehavioral Study - Headcap | Euthanized due to experimental study | No | No | Dz Corr, Bldwk, Term Wt, Prog Wt, Fecal Bio |

* Animals <2 years of age at death were excluded from body weight and bone-specific biomarker analyses.

M = male; F = female; U = Unknown; ND = Nondiagnostic

Dz Corr = disease correlation analysis

Bldwk = complete blood count and serum chemistry panel analyses (Figures 1A & S1A)

Term Wt = terminal body weight analysis (Figures 1B, S1B, & S1C)

Prog Wt = progressive body weight analysis (Figures 2A & 2B)

Rad = radiograph analysis (Figures 3A & 3B)

Ser Bio = serum biomarker analysis (Figures 3C, S2A, S2B, & S2C)

Fecal Bio = fecal biomarker analysis (Figures S2D & S2E)
