# Supplementary material for: Serum Albumin and Body Weight as Biomarkers for the Antemortem Identification of Bone and Gastrointestinal Disease in the Common Marmoset
Source: PLoS One. 2013 Dec 6;8(12):e82747. doi: 10.1371/journal.pone.0082747 (PMC3855796; doi:10.1371/journal.pone.0082747)
Supplement: Table S2 — Biomarkers found to not cross react with marmoset samples. (DOC) [file pone.0082747.s004.doc]

**Table S2: Biomarkers found to not cross react with marmoset samples**

| **Marker** | **Sample Type** | **Description** | **Company** |
| --- | --- | --- | --- |
| Anti-Saccharomyces cerevisiae Antibody (ASCA) | Serum | Antibody against yeast; marker for inflammatory bowel disease when used in tandem with pANCA | ALPCO Diagnostics, Salem, NH |
| Anti-Tissue Transglutaminase Antibody | Serum | Antibody produced as a reaction to wheat; marker of celiac disease replacing antigliadin antibody | ALPCO Diagnostics, Salem, NH |
| Osteocalcin | Serum | Protein secreted by osteoblasts; marker of bone formation | Immunodiagnostic Systems, Scottsdale, AZ |
| Perinuclear Anti-Neutrophil Cytoplasmic Antibody (pANCA) | Serum | Antibody against neutrophils; marker for inflammatory bowel disease when used in tandem with ASCA | ORGENTEC Diagnostika GmbH, Mainz, Germany |
| Serotonin | Fecal | Marker of gastrointestinal hypermotility secreted by gut enterochromaffin cells | ALPCO Diagnostics, Salem, NH |
