# Supplementary material for: Serum Albumin and Body Weight as Biomarkers for the Antemortem Identification of Bone and Gastrointestinal Disease in the Common Marmoset
Source: PLoS One. 2013 Dec 6;8(12):e82747. doi: 10.1371/journal.pone.0082747 (PMC3855796; doi:10.1371/journal.pone.0082747)
Supplement: Table S3 — Bloodwork parameters examined in identifying disease in marmosets. (DOC) [file pone.0082747.s005.doc]

**Table S3: Bloodwork parameters examined in identifying disease in marmosets**

| **Parameter** | **Unit** | **Combined Disease versus**  **No Disease** | | | **Bone Disease versus**  **No Bone Disease** | | | **GI Disease versus No GI Disease** | | |
| --- | --- | --- | --- | --- | --- | --- | --- | --- | --- | --- |
|  |  | **Non-affected** | **Affected** | ***P* value** | **Non-affected** | **Affected** | ***P* value** | **Non-affected** | **Affected** | ***P* value** |
| **Hematocrit** | % | 33.7  (33.7, 33.7) | 34.0  (22.1, 42.0) | I.N. | 35.9  (14.1, 72.2) | 34.8  (22.1 44.7) | 0.88 | 40.1  (33.0, 46.1) | 34.8  (14.1, 50.4) | 0.082 |
| **Platelet Count** | K/uL | 149  (149, 149) | 163  (34, 529) | I.N. | 203  (11, 686) | 264  (34, 529) | 0.86 | 303  (149, 376) | 257  (11, 868) | 0.43 |
| **Aspartate Amino-transferase** | U/L | 180  (86, 274) | 174  (67, 727) | 0.76 | 183  (86, 2964) | 164  (67, 727) | 0.75 | 189  (86, 978) | 174  (67, 5532) | 0.99 |
| **Alkaline Phosphatase*** | U/L | 131  (96, 165) | 150  (81, 1150) | 0.55 | 132  (81, 227) | 167  (81, 1150) | 0.19 | 132  (96, 665) | 143  (45, 1150) | 0.76 |
| **Total Protein** | g/dL | 7.0  (6.3, 7.7) | 5.6  (5.0, 6.8) | 0.12 | 7.6  (6.3, 8.0) | 6.0  (5.0, 7.0) | 0.0051** | 6.5  (4.9, 8.7) | 6.1  (4.6, 7.8) | 0.26 |
| **Serum Calcium*** | mg/dL | 8.9  (8.8, 9.0) | 8.2  (4.1, 9.3) | 0.16 | 9.1  (8.0, 11.0) | 8.2  (4.1, 9.3) | 0.011** | 8.8  (7.1, 10.6) | 8.2  (2.8, 10.9) | 0.12 |
| **Corrected Calcium*** | mg/dL | 8.7  (8.2, 9.2) | 9.2  (4.9, 10.8) | >0.99 | 9.3  (8.2, 10.0) | 9.2  (4.9, 10.8) | 0.26 | 9.2  (8.0, 10.0) | 9.1  (4.0, 11.2) | 0.46 |

Data presented as median values with parentheses following denoting minimum and maximum values

I.N. = insufficient number of subjects for statistical analysis

*Animals less than 2 years of age at death were excluded from analyses of these parameters

**Denotes significant *P* value (*P* < 0.5)
